# Sudden shortening of the paced AV delay: is this normal pacemaker function?

**DOI:** 10.1007/s12471-014-0645-6

**Published:** 2015-01-14

**Authors:** Arnold Pinter, Paul Dorian

**Affiliations:** St. Michael’s Hospital and University of Toronto, 30 Bond Street, M5B 1W8 Toronto, ON Canada

The 200 ms paced AV delay is equal to the native AV conduction time, which leads to the phenomenon called ventricular pseudofusion as the ‘paced QRS’ morphology is identical to the native QRS morphology (QRS complexes marked with an asterisk, Fig. 1) [1]. This is an important observation for two reasons. First, the pacemaker should be reprogrammed to an extended paced AV delay to avoid unnecessary ventricular pacing. Secondly, the presence of ventricular pseudofusion is the clue to answer the question about the short paced AV delay of the third beat.

**Fig. 1:**
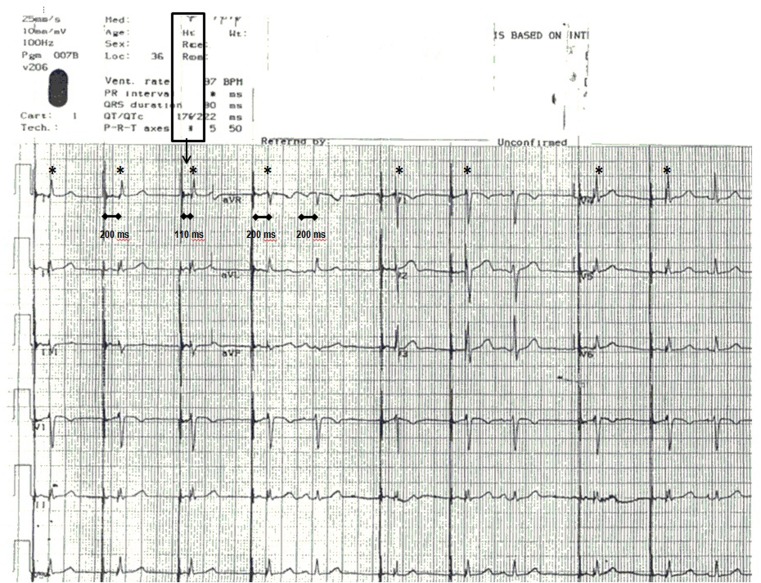
Postoperative standard 12-lead ECG (1 mV/10 mm, 25 mm/s paper speed). The pseudofusion QRS complexes are marked with an asterisk. See text for details

There are various pacemaker features that can shorten the paced AV delay from the set parameter, e.g. ventricular safety pacing [2]. However, in this case, there is no need for knowledge about pacemaker features.

If this was a normal pacemaker feature, we should see fully paced QRS morphology when the pacemaker shortens the AV delay to 110 ms (third beat on Fig. 1). The only potential physiological explanation is a junctional ectopy, occurring exactly on time with the ventricular pacing spike, exactly at the time the paced AV delay is shortened, which is extremely unlikely. Therefore, this has to be an artifact.

When one examines the printed text above the ECG, the letters are squeezed together right above the part of the tracing with the shortened paced AV delay (rectangular frame in Fig. 1). The ECG machine intermittently stopped rolling the paper for about 90 ms at a time, effectively cutting out 90 ms segments from the ECG tracing. Since the paper had a pre-printed grid, this unusual artifact was not easily detectable.

## Conflict of interest

None of the authors have any conflict of interest related to this report.
